# SEB-YOLO: An Improved YOLOv5 Model for Remote Sensing Small Target Detection

**DOI:** 10.3390/s24072193

**Published:** 2024-03-29

**Authors:** Yan Hui, Shijie You, Xiuhua Hu, Panpan Yang, Jing Zhao

**Affiliations:** 1 School of Computer Science and Engineering, Xi’an Technological University, Xi’an 710021, China; youshijie@st.xatu.edu.cn (S.Y.); huxiuhua@xatu.edu.cn (X.H.); yangpanpan@st.xatu.edu.cn (P.Y.); zhaojing@st.xatu.edu.cn (J.Z.); 2 State and Provincial Joint Engineering Laboratory of Advanced Network, Monitoring and Control, Xi’an 710021, China

**Keywords:** small target detection, YOLOv5, feature fusion, attention mechanism

## Abstract

Due to the limited semantic information extraction with small objects and difficulty in distinguishing similar targets, it brings great challenges to target detection in remote sensing scenarios, which results in poor detection performance. This paper proposes an improved YOLOv5 remote sensing image target detection algorithm, SEB-YOLO (SPD-Conv + ECSPP + Bi-FPN + YOLOv5). Firstly, the space-to-depth (SPD) layer followed by a non-strided convolution (Conv) layer module (SPD-Conv) was used to reconstruct the backbone network, which retained the global features and reduced the feature loss. Meanwhile, the pooling module with the attention mechanism of the final layer of the backbone network was designed to help the network better identify and locate the target. Furthermore, a bidirectional feature pyramid network (Bi-FPN) with bilinear interpolation upsampling was added to improve bidirectional cross-scale connection and weighted feature fusion. Finally, the decoupled head is introduced to enhance the model convergence and solve the contradiction between the classification task and the regression task. Experimental results on NWPU VHR-10 and RSOD datasets show that the mAP of the proposed algorithm reaches 93.5% and 93.9%respectively, which is 4.0% and 5.3% higher than that of the original YOLOv5l algorithm. The proposed algorithm achieves better detection results for complex remote sensing images.

## 1. Introduction

With the rapid advancement of space remote sensing technology, there is a notable increase in the quantity of high-resolution remote sensing images. This surge significantly propels the research in remote sensing image target detection. The purpose of remote sensing image object detection is to precisely identify the location of specific objects within the target image and categorize them. Common targets include aircraft, vehicles, ships, and others. Remote sensing image detection technology plays a crucial role in both military and civilian domains, including applications such as port and airport flow detection, traffic management, and maritime rescue. Limited by data and hardware conditions, traditional remote sensing image detection methods usually focus on manual feature extraction and description, such as the histograms of oriented gradients [1], visual saliency detection [2] and scale-invariant feature transform [3] and other methods. However, the traditional methods have some shortcomings, such as a lack of learnable ability, weak transfer ability and cumbersome design. Usually, it can not be used well in large-scale remote sensing images. In recent years, with the continuous development of deep learning, significant results have been achieved in image segmentation, object detection and other fields, which makes more scholars apply deep learning methods to remote sensing image detection.

Unlike traditional detection methods that require hand-crafted feature descriptors, deep learning-based detectors rely on learnable high-level semantic information to abstract objects. Existing object detection models based on deep learning are generally divided into two groups. The first group includes two-stage algorithms that rely on candidate regions. These algorithms generate potential regions [4,5] and then perform classification and position regression [6,7], achieving high-precision object detection. Representative algorithms in this category include R-CNN [8], Faster R-CNN [9], Mask R-CNN [10], and Sparse R-CNN [11]. While these algorithms boast impressive accuracy rates, their slower processing speeds hinder real-time detection across all devices. On the other hand, the second group comprises single-stage object detection networks rooted in regression. These algorithms directly forecast the position and class of objects from input images using a single network, bypassing the intricate process of generating candidate regions. This streamlined approach results in significantly faster detection speeds. The main representative networks in this category include SSD [12] and the YOLO [13,14,15,16,17,18] series. These methods have achieved good results on natural image datasets such as MS COCO [19] and PASCAL VOC [20]. Among them, the YOLO series of single-stage detection algorithms is widely used. Currently, YOLOv5 [21] strikes a balanced performance within the YOLO series. However, when these methods are applied to the recognition and detection of remote sensing targets with more complex backgrounds, the effect is not very ideal. The primary rationale is that remote sensing images are captured by sensors mounted on aerospace and aviation equipment. Consequently, the majority of remote sensing images exhibit a broad imaging scope, intricate backgrounds, and an imbalanced distribution of foreground objects. In addition, objects in remote sensing images are always small, and small objects often lack enough appearance information to distinguish them from complex backgrounds compared to large/medium objects. Finally, the modeling location of cellular neural networks limits their ability to capture global context information in remote sensing images [22]. In general, CNN-based object detection algorithms are difficult to be directly applied to detect small objects in remote sensing images. How to deeply mine the target features of small targets in remote sensing images, further improve its detection accuracy, and reduce the rate of missed detection and false detection has become an urgent problem to be solved.

Therefore, this paper introduces an enhanced SEB-YOLO algorithm structure based on YOLOv5 for small object detection in remote sensing images. Firstly, the non-strided convolution is used to reconstruct the backbone network to reduce the feature loss caused by network downsampling as much as possible, enhance the ability of the model to capture complex details, and retain more global features. In order to improve the adaptability of the model to images with different resolutions, we design a pooling module combined with an attention mechanism at the end of the backbone network. Then, in order to solve the problem of insufficient feature fusion of the original neck network, we introduce Bi-FPN [23] and adopt the bilinear interpolation upsampling operation, which enhances the feature extraction ability of the network by learning the weights of different input features while preserving the pixel relationship of the original feature map. Finally, the decoupling head was used to replace the detection head in the model, and the classification problem and regression tasks in the detection head were calculated separately, which accelerated the convergence speed of the model and further improved the performance of target detection in remote sensing images.

Overall, the contributions of this work can be summarized as follows:

- The reconstructed backbone network enhanced the ability of the model to capture complex details because of the addition of the non-strided convolution module.
- A pooling module combined with an attention mechanism is designed and applied to the last layer of the backbone network, which improves the inference speed while improving the accuracy.
- A weighted bidirectional feature pyramid network incorporating bilinear interpolation upsampling is proposed to improve the detection performance of objects of different scales, especially for small objects.
- The original head structure in YOLOv5 is replaced with the decoupled head to improve the overall performance of the model.

## 2. Related Work

### 2.1. Related Improved Algorithms

At present, two-stage object detection algorithms generally have high detection accuracy but slow speed. Therefore, Redmon et al. proposed YOLO [24], YOLOv2 [25], YOLOv3 [26] and other single-stage algorithms, which discard the candidate box generation stage and directly perform target classification and regression operations, improving the real-time detection speed of the target detection algorithm. Bochkovskiy et al. proposed YOLOv4 [27], the neck part of the YOLOv4 model consists of an SPP (Spatial Pyramid Pooling) block [28,29] and a feature aggregation block. SPP block increases the receptive field and extracts the most important features of the CSPDarknet53 backbone output feature map. Feature aggregation block consists of a modified Path Aggregation Network (PANet) [30], which is an advanced version of a feature pyramid network (FPN) [31], which is used in YOLOv3. Glenn et al. proposed YOLOv5, which adds some training skills on the basis of YOLOv4. The most prominent is the introduction of the focus structure in the backbone network to reduce the number of model parameters and improve the utilization rate of the model. Compared with images in general object detection tasks, remote sensing images have many characteristics, such as complex backgrounds, small and densely arranged targets, and large continuous changes of scale.

The general object detection methods outlined above, which rely on deep learning, face challenges when applied to remote sensing image detection. Consequently, it becomes essential to tailor and optimize the model structure based on the unique characteristics of remote sensing images to ensure both high precision and speed. Addressing the intricate backgrounds often present in remote sensing images, Su et al. [32] devised a feature pyramid structure grounded in scale stratification. They also introduced a central regression approach incorporating distance constraints, thereby enhancing the model’s suitability for detecting objects in remote sensing images amidst complex backgrounds. Liu et al. [33] optimized the darknet Resblock in YOLOv3 by introducing convolutions in the early layers to enhance spatial information. Nevertheless, with the progression of time, the darknet framework has started to show signs of being somewhat outdated. In a more recent development, Luo et al. [34] elevated detection performance by refining the feature extraction module within the YOLOv5 backbone network. Their improvements were substantiated through rigorous validation using a substantial dataset. Zhang et al. [35] improved the pyramid pooling module and added the attention mechanism on the basis of YOLOv5, and introduced the Bi-FPN structure to enhance multi-scale feature fusion, and the improved algorithm achieved better detection results on complex remote sensing images. Based on the convolutional attention module, Xie et al. [36] proposed the simultaneous convolutional attention module and applied it to the YOLOv5 backbone network, which brought significant improvement to the accuracy of the network.

However, the above methods do not improve the detection results of small targets. To solve this problem, Jiang et al. [37] proposed an optimization model of a deep neural network, which considers the characteristics of the target in the image and rethinks the construction of the original input data, and improves the detection effect of small-scale rectangular targets to a certain extent. Zhou et al. [38] innovatively introduced the Combine Attention and Receptive Fields Block (CARFB), a module designed for receptive field feature extraction. Additionally, they incorporated the DyHead dynamic target detection head to improve the model’s ability to perceive smaller objects. Liu et al. [39] proposed an improved YOLOv5 method based on a needed residual transformer (NRT-YOLO). NRT-YOLO incorporates an additional prediction head and integrates innovative nested residual transformer and nested residual attention modules. Notably, this model exhibits proficient capabilities in detecting smaller objects within samples. Zhao et al. [40] redesigned the convolutional block by adding ECA attention [41] and introduced the Swin Transformer [42] network structure and CA attention [43] in the feature fusion stage to enhance the global perception ability of the network and improve the detection accuracy of small targets. Xu et al. [44] proposed a locality-aware backbone network based on Swin Transformer, which optimized the edge detail segmentation and improved the detection accuracy of small objects by designing a cascade network framework with spatial attention interlaced execution. The above methods introduce the Transformer structure to extract features, and the accuracy of small target detection is greatly improved, but the model inference time is prolonged and the detection speed is reduced. There are many kinds of targets in remote sensing images, so there are problems that the target scales are different and the continuous change is large. To solve this problem, Ye et al. [45] proposed an improvement based on the EfficientDet [46] model, introducing a spatial attention model into the construction of the adaptive attention fusion mechanism, and using the fusion factor to fuse parallel spatial and channel attention in an optimal proportion, achieving good results in multi-scale remote sensing image detection. Li et al. [47] proposed an automatic object detection model, AFF-CenterNet, which fused the feature-sharing structure of parallel layers with dilated convolution and attention constraint for feature fusion, effectively improving the detection effect of multi-scale remote sensing aircraft. However, the research object of the above methods is single, the generalization is weak, and there are still many false detections and missed detections in the detection results.

In summary, the existing algorithms have achieved certain results in remote sensing image detection tasks, but they still fail to solve the problems of complex background information, small target information and the loss of small target information transmission in the process of convolutional feature extraction and downsampling operation in remote sensing images. The recent YOLOv7 and the same series of models are larger than YOLOv5, contain more convolutional layers and parameters, and therefore require more computational resources and time to train and reason. YOLOv5 is faster, can process more images with the same computational resources, and is more suitable for tasks in the field of real-time object detection and remote sensing.

Given YOLOv5’s notable attributes, such as high reliability, stability, ease of deployment and training, along with being one of the most accurate single-stage detection algorithms, it is selected as the benchmark network for this study. The subsequent enhancements in this research focus on optimizing feature extraction and fusion methods, along with integrating attention mechanisms. The goal is to address the specific challenge of detecting small targets in contemporary remote sensing images.

### 2.2. Introduction to the YOLOv5 Network

YOLOv5 is one of the representative algorithms of the one-stage detection method. YOLOv5s has the characteristics of a small model and fast detection speed. Its network structure mainly consists of four parts: input, backbone network, neck network, target location and category prediction layer.

At the input part, Mosaic data enhancement is used to enrich the dataset, and four images are randomly selected for scaling, cropping and splicing. When the image size is different, adaptive image scaling can be performed to reduce the amount of calculation and improve the detection speed. The backbone network is configured with the CSPDarkNet architecture. In the initial layer, the focus structure from the previous version is substituted with a 6 × 6 convolutional layer. In the new version of YOLOv5, the Spatial Pyramid Pooling structure (SPP) has been adapted to SPPF, incorporating a MaxPool layer in sequence to ensure efficacy and enhance detection speed. The neck network adopts the FPN + PAN structure, where the FPN layer propagates robust semantic features from top to bottom, and the feature pyramid transmits strong positional features from bottom to top. Parameters from various backbone layers are aggregated to augment the network’s feature extraction capabilities. The Prediction output filters the overlapping region between confidence bounding boxes and other candidate boxes, ultimately predicting objects of varied sizes on different-sized feature maps. Following non-maximum suppression, the prediction category with the highest confidence score is then output.

## 3. Improved YOLOv5 Model

**Figure 1:**
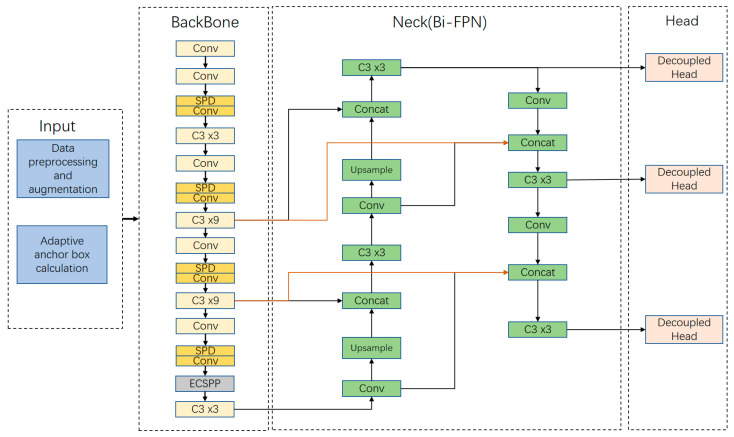
The architecture of the SEB-YOLO.

Detecting small targets in remote sensing images presents challenges in achieving effective feature extraction and multi-scale feature target fusion. The original YOLOv5 model struggles to meet these detection requirements, resulting in low accuracy and a high rate of missed detections. In response, the SEB-YOLO network model illustrated in Figure 1 has been designed. The neck part uses the improved Bi-FPN structure, and the red line represents the difference between the feature fusion path of the improved Bi-FPN and the original YOLOv5 neck network.

In this paper, the Max pooling and the convolution with a step size of 2 in the original network are discarded, and a non-strided convolution module is used to reconstruct the backbone network, which minimizes the feature loss caused by network downsampling and retains more global features. Secondly, the last layer of the backbone network is replaced by the ECSPP module designed by us, which helps the network to better identify and locate the target and enhances the performance of target detection. In addition, an improved feature fusion method combining bidirectional feature pyramid and bilinear interpolation upsampling method is used in the neck network to improve bidirectional cross-scale connection and weighted feature fusion to further enhance the features of small targets and reduce the loss of small target feature information and position information. Finally, the detection head in the model is replaced by the decoupling head to distinguish regression and classification, accelerate the convergence speed of the model, and further improve the detection performance of small objects in the image.

### 3.1. Space to Depth and Non-Strided Convolution

In the original network of YOLOv5, there is a large number of strided convolutions, which is a common but flawed design that may lead to the loss of critical fine-grained information in remote sensing images and the reduction in the efficiency of feature representation learning. In an effort to address these limitations and enhance small object detection in remote sensing images, the SPD-Conv [48] is introduced as a substitute for the sstrided convolution. Comprising a space-to-depth conversion layer and a non-strided convolution layer, the SPD-Conv module compresses image pixels, transforming two-dimensional spatial data into a more concise one-dimensional representation. This innovative approach is designed to overcome the limitations of traditional strided convolutions. It aims to enhance the model’s ability to capture intricate details, especially when dealing with small objects in remote sensing images.

**Figure 2:**
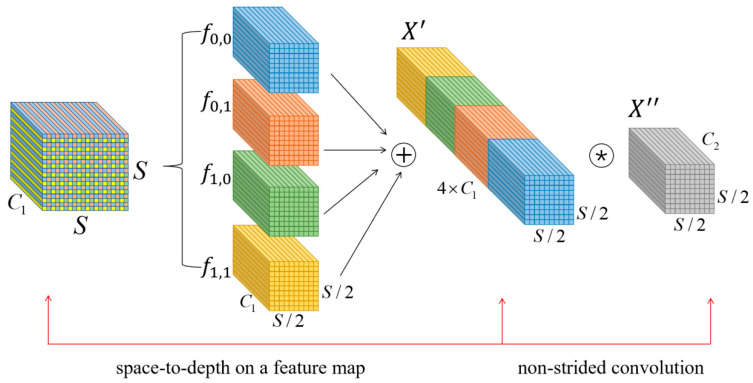
Space to epth and non-strided convolution.

The SPD-Conv operation consists of two steps. Firstly, the feature map of the input image undergoes preprocessing from space to depth; subsequently, the preprocessed feature map is subjected to a standard convolution. In general, given any (original) feature map *X*, a sub-map $f_{x,y}$ is formed by all the entries *X(i, j)* that *i + x* and *j + y* are divisible by scale. Therefore, each sub-map downsamples *X* by a factor of *scale*. Figure 2 give an example when *scale* = 2, where we obtain four sub-maps $f_{0,0}$,$\;f_{0,1}$,$\;f_{1,0}$ and $f_{1,1}$, each of which is of shape ($\frac{S}{2}$, $\frac{S}{2}$, $C_{1}$) and downsamples *X* by a factor of 2. The obtained feature maps are spliced according to the channel direction to obtain the output feature map $X^{\prime }\left(S∕scale,S∕scale,scale^{2}C_{1}\right)$, whose channel number is four times the channel number of the input tensor, and the transformation of spatial depth is completed. After that, the module adds a convolution layer with a $C_{2}$ filter to achieve further transformation of the features, and another non-strided convolution will be performed. The step size of this convolution layer is set to 1, thus preventing non-discriminatory loss of information and retaining all discriminative feature information as much as possible. The final feature map $X^{\prime \prime }\left(S∕scale,S∕scale,C_{2}\right)$ is obtained as shown in Figure 2.

### 3.2. ECSPP Module

The Spatial Pyramid Pooling Fusion (SPPF) module plays a crucial role in extracting feature representations at various scales, enhancing the network’s adaptability to objects of different sizes during the detection process. The input of the SPPF module is the feature map from the backbone network, which can capture semantic information at different scales by dividing the feature map into different subregions and applying pyramid pooling. These feature maps are then fused so that the network can synthetically utilize information at different scales for object detection. This integration significantly enhances the network’s capability to identify objects of varying sizes, consequently elevating overall detection accuracy.

**Figure 3:**
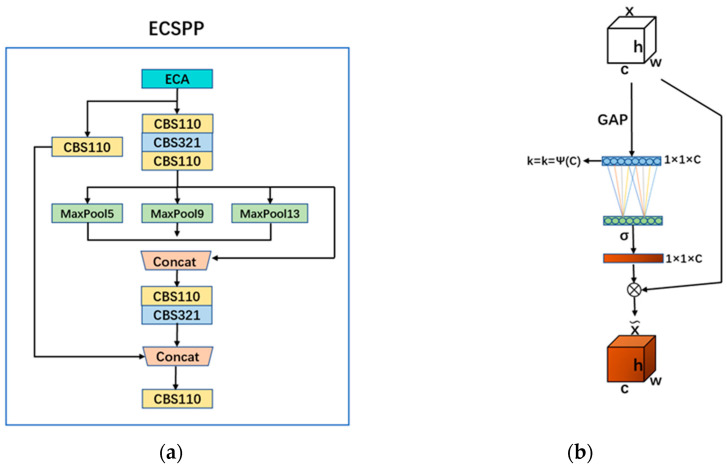
ECSPP module. (**a**) The network structure of ECSPP. (**b**) The network structure of ECA-Net.

In our pursuit of enhancing our model’s adaptability to images of varying resolutions, we introduced a novel fusion of advanced techniques. We merged the Spatial Pyramid Pooling Cross Stage Partial Conv (SPPCSPC) module derived from YOLOv7 with the sophisticated attention mechanism known as the Efficient Channel Network (ECA-Net). This innovative combination was aptly christened the ECSPP (ECA-Net+SPPCSPC) module, and its architectural representation can be observed in Figure 3a. Within the confines of the ECSPP module, we meticulously partitioned the input features into two distinct components: one tailored for convolutional operations and the other tailored to the SPP structure. The harmonious integration of these components was meticulously achieved through the application of the Concat operation. The overarching aim of this design was to significantly alleviate computational overhead, leading to accelerated model inference and improved overall accuracy. Simultaneously, in our unwavering commitment to elevating the efficacy and precision of image information processing, we introduced the ECA-Net attention mechanism at the summit of the ECSPP module. This strategic addition empowers the model to selectively suppress superfluous data emanating from diverse channels, allowing it to intensify its focus on the core regions of interest, ultimately leading to more efficient and accurate image analysis.

As depicted in Figure 3b, the ECA-Net attention mechanism enhances feature discrimination by recalibrating individual channels in feature maps based on local spatial context. It achieves this by employing a lightweight one-dimensional convolution operation across the spatial dimension of feature maps, allowing for adaptive channel re-weighting. It avoids the dimension reduction operation and effectively adjusts the channel feature weight distribution, which enhances the representation ability of the network.

### 3.3. Improvement of Feature Fusion Path

The PANet structure is used for multi-scale feature fusion in the original YOLOv5 architecture, but this fusion method cannot make full use of the features between different scales, resulting in certain detection accuracy limitations. With the deepening of the network, the model becomes more complex, and it is more difficult to extract semantic features. The shallow network has a higher discrimination rate, and the extraction of location information will be more accurate. Deep networks have a larger receptive field with more semantic information. In order to optimize the feature fusion at different scales, a more efficient Bi-FPN feature fusion structure is introduced into the neck of YOLOv5, which effectively optimizes the inconsistency of feature information at each scale under multi-scales. The feature pyramid structure with optimized weights is employed to amalgamate feature layer inputs from the YOLOv5 backbone through various techniques, conducting weighted operations that consider the contribution of each input feature layer. This iterative fusion process enhances the resulting feature layer with a wealth of semantic and spatial information. Bi-FPN principle is fast normalized fusion, through ownership of unavailability values divided by the value added and normalization, and the weights of the normalized to [0, 1] reduce the amount of calculation. It is defined as Formula (1).

(1) $$O=\sum_{i}\frac{\omega_{i}I_{i}}{\varepsilon +\sum_{j}\omega_{j}}$$

In Formula (1), where $I_{i}$ is the input feature, O is the output feature, $\omega_{i}$ and $\omega_{j}$ are the learnable weights, the ReLU activation function is used to scale the learnable weights between [0, 1], and ε = 0.001 is a small amount to ensure stable output.

**Figure 4:**
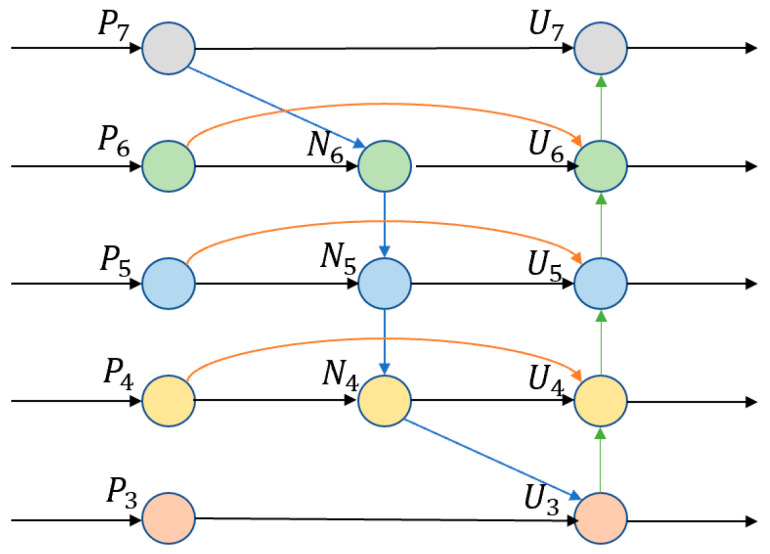
The network architecture of Bi-FPN.

The redundant computation was reduced by reducing some nodes. Skip connections were added to enhance the ability of output layer feature fusion information. The fusion module is formed to further participate in the fusion as a whole. The relationship of each layer is defined as Formula (2):

(2) $$U_{7}=Conv\left(p_{7}\right)\;U_{6}=Conv\left(P_{6}+Resize\left(U_{7}\right)\right)\;U_{5}=Conv\left(P_{5}+Resize\left(U_{6}\right)\right)\;U_{4}=Conv\left(P_{4}+Resize\left(U_{5}\right)\right)\;U_{3}=Conv\left(P_{3}+Resize\left(U_{4}\right)\right)$$

where Conv is a deep separable convolution operation, and Resize is an upsampling or downsampling operation. $P_{i}$ is the feature map extracted by the backbone and $U_{i}$ is the output feature map. By constructing a bidirectional feature pyramid network, the detection accuracy can be improved and the calculation amount of the model can be reduced. The network structure of Bi-FPN is shown in Figure 4.

In the YOLO v5 model, the nearest neighbor interpolation method is utilized for the upsampling of the feature map in the feature fusion part. The nearest neighbor interpolation up-sampling method does not calculate the value of the point in the pixel matrix of the new interpolated feature map and directly finds the corresponding pixel value in the position information of the original feature image, so the sampling speed is fast. However, this sampling method easily destroys the pixel relationship of the original feature map, resulting in image distortion, the loss of small target feature and location information, and increasing the missed detection and false detection rate of small targets. To solve this problem, this paper integrates the bilinear interpolation upsampling operation into the Bi-FPN structure to realize the upsampling of the feature map and expand the scale of the feature map.

When calculating the pixel value of the new feature map, the bilinear interpolation upsampling selects four points from the original image and interpolates them in two vertical directions, which is the extension of the single linear interpolation. Through the cubic interpolation in two directions, the sampling method ensures the relationship between the original feature map pixels while upsampling the feature map, which can improve the clarity of the sampled feature map, reduce the loss of small target feature information and location information, and reduce the missed detection and false detection rate of small target detection. The principle of this sampling method is as follows.

**Figure 5:**
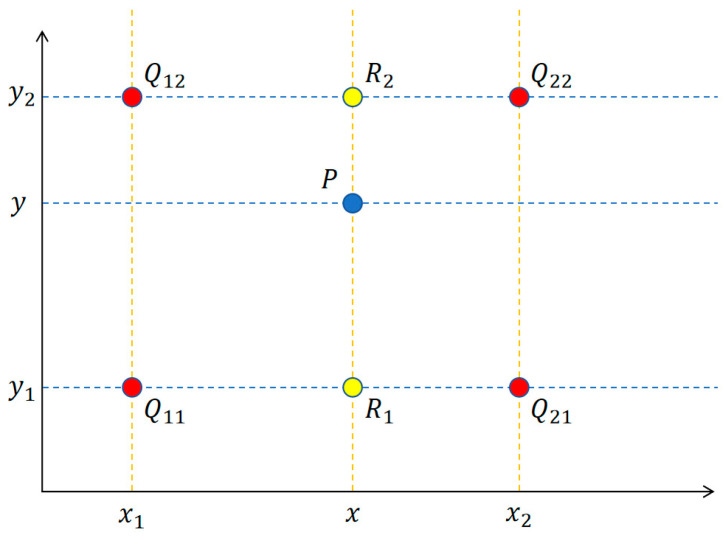
Bilinear interpolation.

Suppose that the pixel values of four points selected in the original image are $Q_{11}$($x_{1}$,$y_{1}$), $Q_{21}$($x_{2}$,$y_{1}$), $Q_{12}$($x_{1}$,$y_{2}$), $Q_{22}$($x_{2}$,$y_{2}$). First, perform linear interpolation in the *x*-axis direction to obtain the interpolation results of $R_{1}$(x,$y_{1}$) and $R_{2}$(x,$y_{2}$); then, perform linear interpolation in the *y*-axis direction to obtain the interpolation result of point P(x,y). In Figure 5, data points with known values are highlighted in red and denoted by the letter Q. The data points slated for interpolation are marked in blue, represented by the letter P. Points in the intermediate transition are distinguished in yellow and designated by the letter R. The four red points Q are points in the original image, and the blue point P represents the projection of the pixel point from the target image onto the original image. These four red points Q are the nearest points around the projection point P. By using these four red points Q, the pixel value of the projection point P can be calculated. This process determines the pixel values of the points on the target image, the detailed operation steps are shown in Formulas (3)–(6). First, we interpolate twice in the *x*-axis direction, and Formulas (3) and (4) can be utilized to calculate the values of $R_{1}$ and $R_{2}$, respectively.

(3) $$f\left(R_{1}\right)\approx \frac{x_{2}-x}{x_{2}-x_{1}}f\left(Q_{11}\right)+\frac{x-x_{1}}{x_{2}-x_{1}}f\left(Q_{21}\right)$$

(4) $$f\left(R_{2}\right)\approx \frac{x_{2}-x}{x_{2}-x_{1}}f\left(Q_{12}\right)+\frac{x-x_{1}}{x_{2}-x_{1}}f\left(Q_{22}\right)$$

The value of the target point *P* is inserted by linear interpolation in the *y*-axis direction.

(5) $$f\left(P\right)\approx \frac{y_{2}-y}{y_{2}-y_{1}}f\left(R_{1}\right)+\frac{y-y_{1}}{y_{2}-y_{1}}f\left(R_{2}\right)$$

Finishing available:

$$f\left(x,y\right)\approx \frac{f\left(Q_{11}\right)}{\left(x_{2}-x_{1}\right)\left(y_{2}-y_{1}\right)}\left(x_{2}-x\right)\left(y_{2}-y\right)+\frac{f\left(Q_{21}\right)}{\left(x_{2}-x_{1}\right)\left(y_{2}-y_{1}\right)}\left(x-x_{1}\right)\left(y_{2}-y\right)+$$

(6) $$\frac{f\left(Q_{12}\right)}{\left(x_{2}-x_{1}\right)\left(y_{2}-y_{1}\right)}\left(x_{2}-x\right)\left(y-y_{1}\right)+\frac{f\left(Q_{22}\right)}{\left(x_{2}-x_{1}\right)\left(y_{2}-y_{1}\right)}\left(x-x_{1}\right)\left(y-y_{1}\right)$$

### 3.4. Improved Detection Head

In YOLOv5, the original prediction layer involves resizing the input image to create a feature map of the same dimensions as the input image for subsequent object detection. However, this method has some associated challenges. For example, for large-size objects, detection on smaller feature maps may lead to accuracy degradation. For small-size objects, detection on larger feature maps may lead to too low detection accuracy. In the classification and regression tasks of object detection, spatial misalignment occurs because of the different points of interest for each task. Classification emphasizes the similarity of the extracted features to existing categories, while regression concentrates on positional coordinates to refine bounding box parameters. Using the same feature map for both tasks can significantly diminish the overall effectiveness. In order to solve the contradiction between the classification task and the regression task, the improved decoupled head uses multiple feature maps of different sizes for object detection, so that it can better detect objects of various sizes.

The decoupled head, initially introduced in YOLOX, has since found widespread application in various object detection algorithm tasks. This paper introduces the decoupled head at the prediction layer to enhance detection accuracy, expedite network convergence, and improve overall detection performance. In particular, the decoupled head reduces the input image to multiple feature maps of varying sizes and subsequently conducts object detection separately on these feature maps. This approach helps mitigate the aforementioned issues. The decoupled head also has the advantage that it can regulate the balance between accuracy and efficiency by adjusting the size of each feature map.

**Figure 6:**
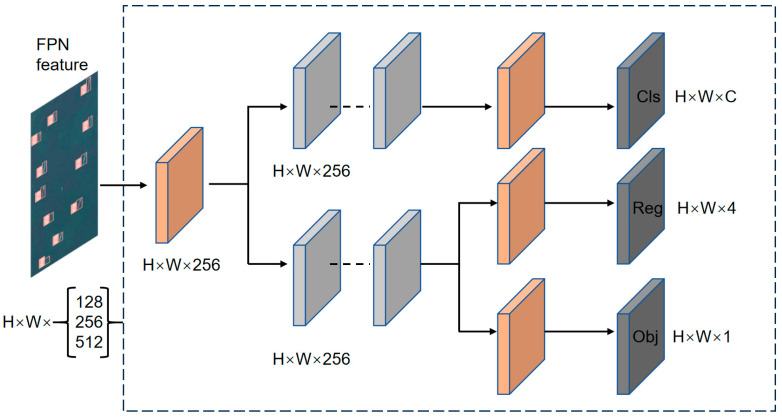
The decoupled head.

Figure 6 illustrates the structure of the decoupled head. Initially, a 1 × 1 convolution is employed to reduce dimensionality. Subsequently, two parallel branches for classification and regression utilize two 3 × 3 convolutions each. In the classification branch, a 1 × 1 convolution is applied for classification purposes. Meanwhile, in the regression branch, a 1 × 1 convolution is employed in each of the two parallel branches dedicated to localization and confidence operations, respectively. The anchor-based method is then utilized to extract the target frame, followed by a comparison with labeled ground truth to assess the disparity between the two. Ultimately, this process yields the detection result.

## 4. Experimental Results and Analyses

### 4.1. Experimental Dataset

The dataset employed is the NWPU VHR-10 [49], a georemote sensing dataset for space object detection, released by Northwestern Polytechnical University. It includes 650 images with objects and 150 background images, resulting in a total of 800 images and 3896 object instances. The dataset is annotated and verified and can be easily used for the training and testing of object detection algorithms in remote sensing images. This dataset contains a large number of small-size objects, such as airplanes, oil storage tanks, vehicles, etc., accounting for up to 44.3% of the total. These targets are affected by factors such as image shooting angle, occlusion and illumination, and have different sizes and shapes in remote sensing images, which have reference values for the test of remote sensing image detection algorithms.

**Figure 7:**
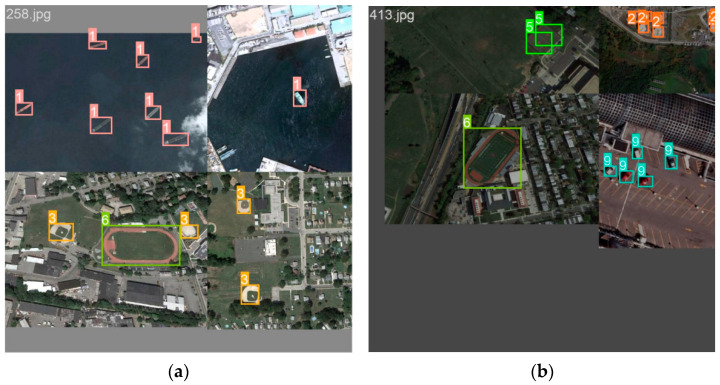
Mosaic data augmentation. (**a**) Augmentation including ships. (**b**) Augmentation including vehicles.

In this paper, the training set and the test set are allocated according to the ratio of 4:1 among the 650 images with targets in the dataset. In the data reading part, image scaling, cropping, flipping and Mosaic data augmentation are used. Mosaic is randomly selected from each of the four images through translation operations such as cutting, combined into an image inside training, on the basis of the original expanded dataset. The data augmentation effect is shown in Figure 7.

### 4.2. Experimental Environment and Metrics

The training and development of this experiment is based on the Pytorch framework under the Linux server with the Ubuntu system, and the specific experimental environment is shown in Table 1.

The experiment was trained, verified and tested under the same environment. All training was from scratch, initialized by the weights trained on the COCO dataset. Experimental hyperparameters: training resolution 1280 × 1280; batch size is 8; the number of epochs is 300. The optimizer is stochastic gradient descent (SGD) and the initial learning rate is 0.01. Stochastic gradient descent was used to optimize the model, and the default values of YOLOv5 were used for the rest of the hyperparameters.

The target detection accuracy of remote sensing images is calculated by the localization accuracy and classification accuracy of the prediction results. In order to effectively verify the performance of the model, this study uses the average precision (mAP), precision (P), recall (R), floating-point calculation (FLOPs) and model volume.

### 4.3. Comparative Experiment

In order to verify the superiority of the algorithm for small object detection model, the same training configuration parameters are used for training, and the parameters of the algorithm and YOLOv5l are compared with some other current mainstream algorithms SSD, Fast-RCNN, YOLOv3, YOLOv4 and the latest YOLOv7 for global and local evaluation.

It can be seen from Table 2 that YOLOv5l has a smaller volume than the two-stage algorithm RCNN and the single-stage SSD, YOLOv3, and YOLOv4 algorithm models. Although the volume of the improved model in this paper is higher than that of YOLOv5l, it is 5.37% higher than that of YOLOv5l in R, and most importantly, 4.0% higher than that of YOLOv5l in mAP. It is much higher than SSD, Faster-RCNN, YOLOv3, and YOLOv4 models. Since the dataset NWPU VHR-10 contains a large number of target categories, the features of different categories are quite different and the scale change is more obvious, which also increases the difficulty of its detection. However, the experimental results of the improved method based on YOLOv5 in this paper are even better than the new YOLOv7 model proposed by the YOLO series, and the mAP is 0.4% higher than that of YOLOv7, which reflects the effectiveness of the improved method. It is worth noting that YOLOv5 trains and deduces faster than YOLOv7. This makes the improved model based on yolov5 more advantageous in some application scenarios, such as mobile devices or resource-constrained systems. Among them, Table 3 shows the evaluation results of different algorithms for different categories of the dataset.

**Figure 8:**
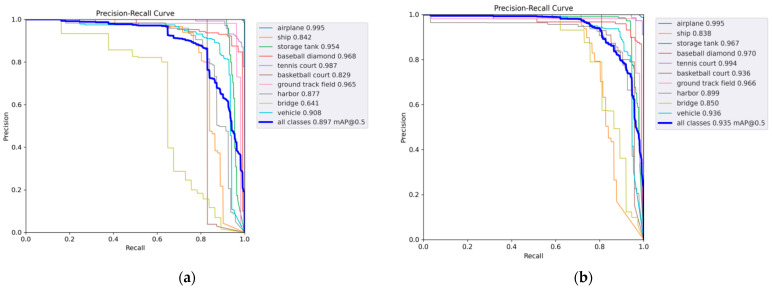
Comparison of the precision–recall curves on NWPU VHR-10 dataset. (**a**) YOLOv5l experimental results. (**b**) SEB-YOLO experimental results.

It can be seen from Table 3 that the average detection accuracy of the algorithm in this paper on the dataset NWPU VHR-10 reaches 93.5%, and the detection effect of eight types of objects is better than that of the baseline network algorithm. In contrast to other algorithms, the algorithm presented in this paper exhibits additional advantages, particularly in multi-class object detection scenarios. The assessment standards for small target detection typically involve airplanes, ships, and vehicles within the dataset. In this paper, the average detection accuracy of these three types of targets is taken as the mAP of small targets, and the small target mAP is 92.3%. Compared with AAB-YOLOv5, Ghost-YOLOv5 and YOLOv5l small object mAP, it is 0.7%, 2.4% and 1.2% higher, respectively. Therefore, the experimental results demonstrate the effectiveness of the proposed algorithm in small object detection. In comparison to other enhanced algorithms, the proposed algorithm also exhibits superior detection performance for baseball fields, basketball courts, tennis courts, and sports fields with substantial scale variations in the dataset. Furthermore, it demonstrates improved detection capabilities for densely populated small targets, such as oil storage tanks, and for challenging scenarios like bridges that are difficult to separate from the background. The precision–recall curve for the improved algorithm is shown in Figure 8.

However, from a single perspective, the average detection accuracy of ships in the experimental data is not very prominent; the main reason may be that ships are relatively different from other target classes, and the detection is relatively difficult in the complex sea background. In addition, the proportion of ship targets in the NWPU VHR-10 dataset is small, resulting in a general training effect. Compared with other algorithms, except for some shortcomings in some categories of objects, the overall object and small object detection achieve the highest mAP.

### 4.4. Ablation Experiment

In order to verify the optimization effect of each improved module on the original network, for this purpose, ablation experiments are conducted, as shown in Table 4. The first improvement is to introduce SPD-Conv to reconstruct the backbone structure of the network, which has a significant improvement in R compared with the original model, and the mAP is effectively improved by 2.9% with a slight increase in model volume. The second improvement is to add the ECSPP module designed at the end of the original backbone network. Compared with the original model, R is increased by 3.6%, and mAP is increased by 1.4%. The third improvement integrates the bilinear interpolation upsampling method and Bi-FPN feature fusion method into the original network. In comparison to the original model, P remains essentially unchanged, resulting in a minimal increase in model size, while achieving a notable improvement in mAP by 2.6%. In the fourth improvement, the detection head of the original network is modified to the improved decoupled head. Compared with the original model, P is increased by 1.0%, R is slightly increased by 4.5%, and mAP is increased by 3.7%. The analysis shows that the above improvements have their own advantages, the fourth improvement has advantages in improving P and R, the first improvement has advantages in improving accuracy, and the third improvement has advantages in model volume.

According to the analysis of other fusion experiments in Table 4, in general, the improved algorithm in this paper has certain advantages in improving P, R and mAP. In general, the proposed algorithm improves the mAP by 4.0% compared with the original network model. However, due to the increase in model complexity, the detection speed will be reduced and the model volume will be increased. Therefore, compared with the original network model, the average detection time and model volume are slightly inferior, but the small increase in time and model volume will not have a substantial impact on the lightweight and real-time performance of the model. At the same time, it is very cost-effective to trade a small increase in detection speed and model volume for a large increase in detection accuracy.

**Figure 9:**
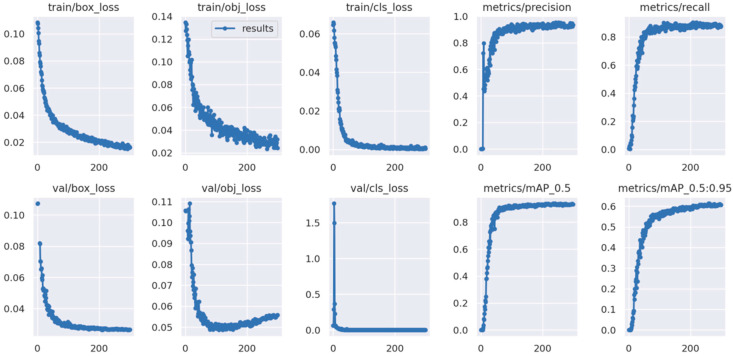
The training results of the improved algorithm on the NWPU VHR-10 dataset.

Figure 9 shows the changes in common metrics during the training of the improved algorithm. From the performance curve of confidence loss during training, we observe that the confidence loss of our method converges relatively swiftly, and the model’s training metrics gradually improve with increasing training iterations. The accuracy, recall and mAP are higher than those of the original YOLOv5 model, and reach a stable state after fewer training times. Through a combination of ablation experiments and verification on the NWPU VHR-10 dataset across various target recognition categories, the following conclusions have been derived.

**Figure 10:**
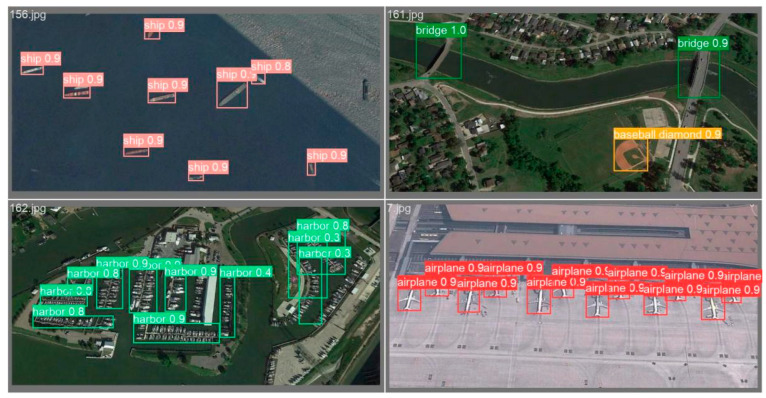
The detection effect of the improved algorithm.

By reconstructing the feature extraction network and incorporating the SPD-Conv module, the loss of crucial fine-grained information in remote sensing images can be minimized, enhancing the efficiency of feature representation learning. This refinement results in greater sensitivity for detecting small objects, facilitating improved categorization and enhancing both detection accuracy and recognition rates. The addition of the ECSPP module, complemented by ECA-Net, has the effect of selecting vital information for the task at hand. This boosts attention to the target area, ultimately enhancing the efficiency and accuracy of image information processing. The use of bilinear interpolation upsampling, combined with the integration of Bi-FPN, proves to be instrumental in improving the extraction of additional semantic information for small targets in remote sensing. This approach effectively reduces the influence of background information on the target, mitigating false detection rates and missed detection rates to a certain extent. The refinement of the network detection layer, specifically through the optimization using the decoupled head, allows for the adjustment of the size of each feature map, thereby striking a balance between accuracy and efficiency. Multiple feature maps of varying sizes are employed, leading to a marked improvement in the overall accuracy and efficiency of YOLOv5. The detection effect of the algorithm after the overall improvement is shown in Figure 10.

### 4.5. Experiment on RSOD Dataset

To validate the universality of the enhanced algorithm proposed in this paper for target detection in remote sensing images and its superior detection capabilities compared to other mainstream algorithms, a comparative analysis is conducted with existing mainstream algorithms. Six models, including typical target detection models such as SSD, Fast-RCNN, YOLOv3, and the recently improved Swin-YOLOv5s based on YOLOv5, are selected for comparative test. The same remote sensing image dataset RSOD was used in the experiment, this dataset was published by Wuhan University in 2015 for the number of criteria for object detection in remote sensing images [50]. There are 976 images and 6950 instances in the dataset, including 446 images of aircraft with 4933 instances, 165 images of oil tanks with 1586 instances, 176 images of bridges with 180 instances, and 189 images of operating fields with 191 instances. The RSOD dataset is the PASCAL VOC format as the specification. To meet the dataset format of YOLO training, the PASCAL VOC format is converted to the YOLO format. From these, 546 images are selected as the training set, 137 images are selected as the validation set, and the remaining 293 images are selected as the test set. In the training process, the SGD algorithm is used to train for 300 epochs, the initial learning rate is set to 0.01, and the step size decay strategy is used to reduce the learning rate. After each epoch, The learning rate is reduced by 10% to avoid the model falling into the local optimal solution in the later training process. Experiments show that this learning rate ensures the rapid convergence of the model in the initial stage, and does not cause the gradient explosion or disappearance. Based on the hardware configuration and the complexity of the model, the batch size is set to 8. In this setting environment, the model can run stably on the hardware and obtain a reasonable training speed. The experimental results are shown in Table 5.

Table 5 shows that the improved method achieves the best mAP value results in RSOD, which is 4.5% higher than the second-best detection effect of YOLOX. Compared with the original YOLOv5l, although the accuracy of the airport category is reduced, the accuracy of other categories and the mAP value are greatly improved. In addition, the mAP value of the proposed method reaches 93.9, while the map value of the Swin-YOLOv5s is only 88.9 compared with the Swin-YOLOv5s, which is the nearest modified based on YOLOv5. The method proposed in this paper does not completely solve the problem of unbalance of small target classes on the model detection accuracy. There is the problem of unbalance of target classes in the data set, which also affects the final precision of the training results. The classification imbalance of small target detection has always been a difficult problem in target detection at this stage, which is also worth further research.

**Figure 11:**
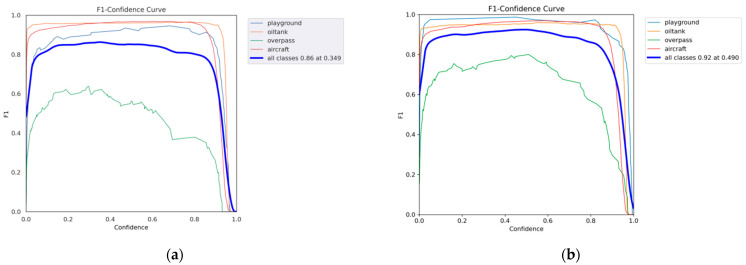
Comparison of the F1–confidence curves on the RSOD dataset. (**a**) YOLOv5l F1–confidence curve. (**b**) SEB-YOLO F1–confidence curve.

Figure 11 shows the F1–confidence curve of the improved algorithm on the RSOD dataset. As can be seen from the figure, the overall robustness of the classification model is relatively high. The detection results of overpass targets are slightly worse than other algorithms because the same type of overpass targets show large differences in appearance due to their different road conditions. Although the accuracy of the algorithm in this paper is sometimes decreased compared with other algorithms, the detection accuracy of most categories of objects is improved.

**Figure 12:**
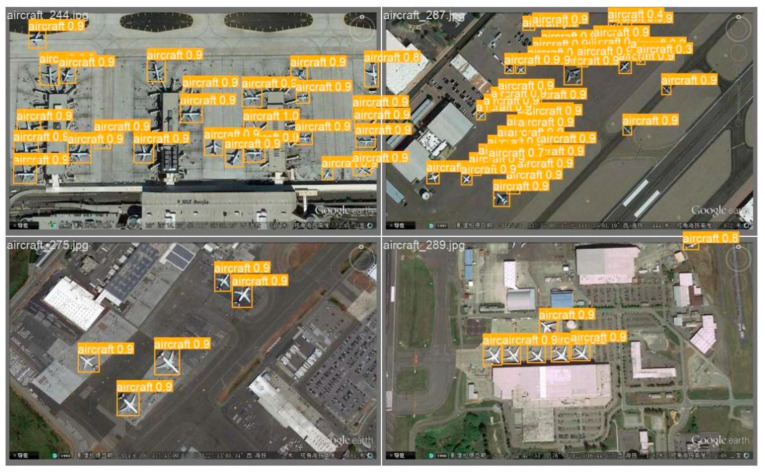
Intensive aircraft target detection.

In general, the improved method has outstanding performance in the detection of playground, aircraft and oil tank targets, which shows the effectiveness of the algorithm for multi-scale targets. Figure 12 shows an example of the detection results of the improved algorithm in this paper on the RSOD dataset, from which it can be seen that the improved network has good adaptability to dense targets and does not lose targets at the edge of the image. The improved network has a good effect on the small aircraft targets with insignificant characteristics.

## 5. Conclusions

The detection and recognition of targets in remote sensing images has very important economic and strategic value in military and civilian aspects. Due to the high complexity of the background of remote sensing images, small targets are dense and the feature information is too little, which leads to great difficulties in the process of small target recognition. In this paper, the feature extraction network is reconstructed and the ECSPP module is added. The original model feature fusion network was improved to Bi-FPN, and the bilinear interpolation upsampling method was used. The decoupling head is introduced in the detection layer. Experiments on NWPU VHR-10 and RSOD data sets show that the mAPs of the proposed algorithm reach 93.5% and 93.9%, respectively, which are 3.9% and 5.3% higher than that of the mainstream YOLOv5 algorithm. Complex scenes have greater interference with small targets. Although the detection accuracy of the improved YOLOv5 model proposed in this paper has been greatly improved, it still cannot accurately identify all small objects in the image, and there are still missing and false detection cases for small objects in occlusion and clustering scenes. Future research on small target detection technology is mainly focused on improving the portability of small target detection technology, making detection technology no longer confined to a special scene, building a small target data set with strong universality, and solving the problem of unbalanced target categories. Future research can continue to consider the application of feature fusion structures in small target detection technology.

## Figures and Tables

**Table 1:** Experimental environment configuration.

Configuration Name	Environmental Parameters
Operating system	Ubuntu5.4
CPU	Intel(R) Xeon(R) Silver 4210CPU @ 2 20GHz
GPU	4xNVIDIA GTX 2080ti 12G
Memory	120 G
Python	3.7
CUDA	10.1
Pytorth	1.8

**Table 2:** Evaluation results of different mainstream algorithms.

Serial Number	Algorithm Name	P/%	R/%	F1-Score/%	Volume/MB	mAP/%
1	SSD	92.3	78.2	84.6	90.6	74.1
2	Faster-RCNN	63.5	90.8	74.7	108	76.5
3	YOLOv3	88.7	86.1	87.3	235	84.4
4	YOLOv4	87.6	89.7	88.6	244	86.8
5	YOLOv5l	93.6	83.8	88.4	89.3	89.5
6	YOLOv7	93.7	86.1	89.7	72	93.1
7	Ours	92.8	88.3	90.5	151.5	93.5

**Table 3:** Evaluation results of different algorithms on different categories.

Targets& mAP	AP/%								
	SSD	Faster RCNN	YOLOv3	YOLOv4	HRSI-DNN [37]	YOLOv5l	AAB-YOLOv5 [35]	Ghost-YOLOv5 [36]	Ours
airplane	90.3	82.8	92.5	94.6	93.0	99.5	95.3	99.4	99.5
ship	72.5	77.6	75.8	79.8	84.5	83.0	91.9	84.0	83.7
storage tank	60.3	52.5	86.1	94.1	87.1	93.7	88.7	99.5	97.2
baseball diamond	87.5	96.4	89.3	95.4	92.8	97.4	95.8	99.2	97.0
tennis court	58.9	62.7	82.7	89.2	82.0	92.4	91.2	93.1	99.4
basketball court	65.2	69.4	75.5	71.5	89.0	82.0	88.5	89.5	93.5
ground track fild	90.3	98.2	88.4	98.7	78.0	96.4	99.5	99.5	96.7
harbor	80.5	82.6	90.2	80.6	76.0	87.2	92.4	95.9	88.7
bridge	77.9	78.8	84.4	95.3	81.0	72.4	85.1	76.7	85.2
vehicle	57.8	63.7	78.6	68.4	84.5	90.8	87.6	86.3	93.6
mAP/%	74.1	76.5	84.4	86.8	84.8	89.5	91.6	92.3	93.5
Small object mAP/%	73.5	74.7	82.3	80.9	87.3	91.1	91.6	89.9	92.3

**Table 4:** The results of ablation experiments.

Baseline Network	SPD-Conv	ECSPP	Improved Neck	Improved Head	P/%	R/%	F1- Score/%	GFLOPs	Parameters	mAP/%
YOLOv5l					93.6	83.8	88.4	109.6	46,563,709	89.5
YOLOv5l	√				92.8	87.9	90.3	165.1	50,741,619	92.4
YOLOv5l		√			92.2	87.4	89.7	130.2	72,265,088	90.9
YOLOv5l			√		93.2	87.3	90.2	110.4	46,825,853	92.1
YOLOv5l				√	94.6	88.3	91.3	122.2	48,812,015	93.2
YOLOv5l	√	√	√	√	92.8	88.3	90.5	199.1	78,791,545	93.5

**Table 5:** Comparison results between the proposed model and other algorithm models on the RSOD dataset.

Method	Accuracy for Each Category %				mAP
	Aircraft	Oil Tank	Overpass	Playground	
SSD	52.1	96.6	56.7	100	76.4
Faster R-CNN	63.1	84.1	76.9	97.8	80.5
YOLOv3	62.2	95.1	70.4	98.6	81.6
YOLOv4	81.3	98.1	71.7	100	87.8
YOLOv5l	98.2	97.9	60.4	98	88.6
Swin-YOLOv5s	90.4	85.8	81.5	97.9	88.9
YOLOX	87.5	95.3	83.6	91.2	89.4
Ours	98.1	98.4	79.6	99.4	93.9

## Data Availability

Data are contained within the article.
